# Microbial Dysbiosis Tunes the Immune Response Towards Allergic Disease Outcomes

**DOI:** 10.1007/s12016-022-08939-9

**Published:** 2022-06-01

**Authors:** Tracy Augustine, Manoj Kumar, Souhaila Al Khodor, Nicholas van Panhuys

**Affiliations:** 1 Laboratory of Immunoregulation, Sidra Medicine, PO BOX 26999, Doha, Qatar; 2 Microbiome and Host-Microbes Interactions Laboratory, Sidra Medicine, Doha, Qatar

**Keywords:** Adaptive immunity, Microbiome, CD4 +, Hygiene, Allergy, Atopy

## Abstract

The hygiene hypothesis has been popularized as an explanation for the rapid increase in allergic disease observed over the past 50 years. Subsequent epidemiological studies have described the protective effects that in utero and early life exposures to an environment high in microbial diversity have in conferring protective benefits against the development of allergic diseases. The rapid advancement in next generation sequencing technology has allowed for analysis of the diverse nature of microbial communities present in the barrier organs and a determination of their role in the induction of allergic disease. Here, we discuss the recent literature describing how colonization of barrier organs during early life by the microbiota influences the development of the adaptive immune system. In parallel, mechanistic studies have delivered insight into the pathogenesis of disease, by demonstrating the comparative effects of protective T regulatory (Treg) cells, with inflammatory T helper 2 (Th2) cells in the development of immune tolerance or induction of an allergic response. More recently, a significant advancement in our understanding into how interactions between the adaptive immune system and microbially derived factors play a central role in the development of allergic disease has emerged. Providing a deeper understanding of the symbiotic relationship between our microbiome and immune system, which explains key observations made by the hygiene hypothesis. By studying how perturbations that drive dysbiosis of the microbiome can cause allergic disease, we stand to benefit by delineating the protective versus pathogenic aspects of human interactions with our microbial companions, allowing us to better harness the use of microbial agents in the design of novel prophylactic and therapeutic strategies.

## Introduction

Atopic diseases such as asthma, hay fever, atopic dermatitis, and food allergies represent the most common forms of allergy and are typically defined by the presence of specific immunoglobulin E (sIgE) in serum or a positive skin prick test for common environmental allergens. Constituting the most prevalent chronic condition of childhood, significant proportions of children develop atopic symptoms in their first year of life. One recent multinational study indicated that 14–28% of infants suffer from atopic dermatitis [1] and rates of recurrent, severe wheezing often used as an early diagnostic marker of heightened risk for the development of asthma have been reported at 16% [2], with some western countries reporting rates of food allergy in excess of 10% at 12 months of age [3]. Increases in the prevalence of these conditions have largely been observed in industrialized countries and have been linked to the modern western diet and lifestyle. Although, there is now also growing evidence of increasing rates of disease in rapidly developing countries, showing a correlation with rising economic growth and changes in diet and lifestyle [4]. Numerous studies indicate that these types of allergic responses often occur in a progressive manner termed the “atopic march,” initially presenting early in infants as a skin allergy or eczema that is linked to an underlying food allergy [5]. Subsequently, many children go on to become sensitized to indoor allergens, such as dust or pet dander and to develop allergic rhinitis and then asthma later in childhood or in their early teenage years [5]. Sensitization to outdoor aeroallergens such as grass and tree pollens typically occurs during the later phases of the atopic march, at a time where sensitization to food allergens may be seen to decrease [6]. The presence of atopy early in life has been shown to significantly increase the risk for development of additional sensitizations, resulting in a progressive form of atopic disease that advances in an additive fashion [7]. Children initially presenting with atopic dermatitis, the most commonly diagnosed form of atopy within the first 6 months following birth show increased risk for the development of asthma and allergic rhinitis, with the incidence of subsequent disease being associated to the severity of the initially diagnosed atopic dermatitis [5]. These findings imply that certain individuals are predisposed to the development of atopic disease, and early age of onset may be indicative of a susceptible phenotype predictive of increased risk for multiple sensitizations [7]. Many risk factors are associated with the onset of atopic disease, including parental history of atopy [8], breast milk vs. formula feeding [9–12], diet [13], air pollution [14], use of antibiotics [15–17], and mode of delivery [18–20], having been well characterized through epidemiological studies. Whereas data describing the mechanisms linking these environmental factors with the aberrant activation of the adaptive immune system that is responsible for the onset of disease have lagged behind.

**Fig. 1:**
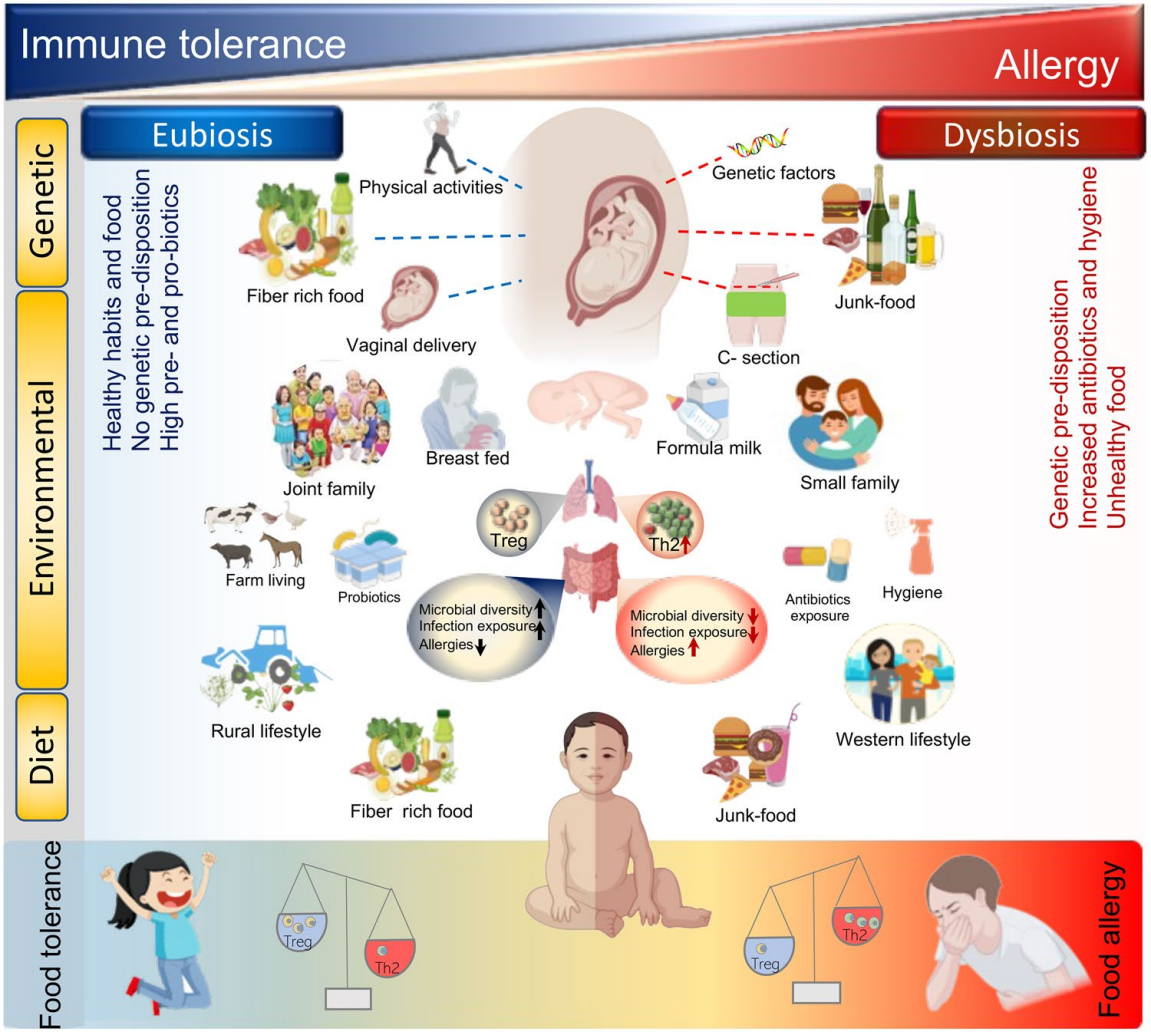
Influences of environmental and microbial interactions on adaptive immune responses and allergic disease. A wide array of factors including, genetic, environmental, and dietary inputs can all potentially modulate the gut immune-microbiome axis and influence the occurrence of allergy. The microbiome in turn modulates the cohort of regulatory cells induced during development and allows for the establishment of a tolerogenic environment, which mediates the suppression of T cells that arise from inflammatory lineages. However, a dysbiosis of the microbiome leads to impairment of this tolerogenic environment leading to development of allergic diseases along with greater expansion in cells of the Th2 inflammatory lineage

The adaptive immune system plays a pivotal role in the development of defense against potential infectious pathogens [21] and as the primary function of the adaptive immune system is to protect against invading pathogens, immune responses generally have an inflammatory effect with potential immunopathological consequences that need to be tightly controlled. To identify potential pathogens, the adaptive immune system requires the ability to distinguish between self and non-self-antigens, whilst simultaneously discerning harmless environmental antigens which can be safely ignored [22]. Occasionally, a failure in the system of checks and balances that is required to maintain immune tolerance occurs, resulting in either autoimmune disease elicited against self-antigens or development of allergic disease against otherwise harmless environmental antigens [23], with studies demonstrating the influence of genetic, developmental, and environmental factors, all contributing to the breakdown of immune tolerance that causes disease [24–27]. Although, significant variations occur, the majority of allergic responses are manifested in early childhood when the immune system is still developing [28–30]. Here, the role of T cells in the initiation of an allergic response has been widely studied, with helper T cells, particularly cells of the Th2 lineage being characterized as the major mediator involved in eliciting allergic responses. Subsequently, it has been determined that a balance between inflammatory Th2 and suppressive Treg cells exists that controls the threshold for allergen sensitization [31] (Fig. 1).

The recent development of “omic” sequencing techniques allowing for the rapid and affordable sequencing of the microbiota has led to studies that have revealed the relevance of the microbiome as a key environmental factor [32, 33]. Allowing us to begin to elucidate how exposure to the natural microbial environment influences the development of the immune system, especially at sites integral for barrier immunity, with new data indicating that host-microbiome interactions during early development play a significant determining role in shaping the immune responses of the host [34, 35].

## Microbial Exposures and the Development of Allergic Disease

### Atopic Dermatitis

Atopic dermatitis, otherwise known as atopic eczema, is an inflammatory skin disorder which mostly develops in childhood and is characterized by itchy eczematous lesions [36]. Atopic dermatitis affects nearly 15–20% of children and 1–3% of adults worldwide [37, 38]. Multiple risk factors contribute to the development of atopic dermatitis, which has a close association with food allergy, particularly in early childhood. The earlier the onset of atopic dermatitis, the higher the risk of food allergy, particularly in relation to peanut, cow’s milk, and hen’s egg allergens [36]. Patients with atopic dermatitis exhibit an increase in the skin pH and lipid deficiency during disease flares, along with the degradation of the skin barrier function [39].

Recently, the skin microbiome has been identified as a critical factor in the development of atopic dermatitis (Table 1) [40–43]. Microbial diversity has been described to have an inverse correlation with atopic dermatitis score (SCORAD), with decreased microbial diversity at sites of disease progression [44]. Specifically, an increase in the relative abundance, particularly of *Firmicutes* (*Gemella* spp., *Staphylococcus aureus* [44] and *Staphylococcus epidermis* and a decrease in the abundance of the phylum *Actinobacteria* (*Dermacoccus* spp.) [45] and Proteobacteria [34] and the genera *Streptococcus*, *Corynebacterium*, and *Cutibacterium* has been associated with atopic dermatitis [34]. Byrd et al. showed that a greater predominance of *Staphylococcus aureus* occur in patients with more severe disease and *Staphylococcus epidermidis* is predominant in patients with less severe disease [46]. *Staphylococcus aureus* strains isolated from patients with atopic dermatitis were enriched with the CC1 strains, whereas the healthy control population was enriched with the CC30 strains [47]. Additionally, topical colonization of mice using strains isolated from patients with atopic dermatitis or controls showed that *Staphylococcus aureus* isolates from patients with more severe disease flares are capable of inducing epidermal thickening and expansion of cutaneous Th2 and Th17 cells, indicating that functional differences in these distinct staphylococcal strains can contribute to the complexity of atopic dermatitis [46].

**Table 1:** Microbial colonization and associations with allergic disease

***Allergy type***	***Location***	***Phylum***	***Family/species/ genera***	***Relative abundance***	***Influence on immune functions***
**Atopic dermatitis**	Skin	*Firmicutes*	*Gemella spp, Staphylococcus aureus *[40, 44, 45]	Increase in abundance	*- S. aureus* produces toxins that accentuate allergic disease through induction of toxin-specific IgE, in addition to activation of various cell types including Th-2 cells, eosinophils, and keratinocytes [44]
					- Superantigens from *S. aureus* can cause an upregulation of cutaneous lymphocyte antigen expression by T cells by stimulating production of IL-12, thereby promoting homing to the skin [44]
					*- S. aureus* can induce a Th2 response through proinflammatory lipoproteins which induce thymic stromal lymphopoietin expression in primary human keratinocytes in a TLR2/TLR6-dependent manner [40]
					- Bacterial challenge assays in keratinocytes and monocyte–derived dendritic cells showed distinct IL-1-mediated innate and Th1-mediated adaptive immune responses with *S. aureus* and *S. epidermidis* [45]
					- Induction of a Th1 polarizing cytokine signature (IL12p70 and IL12p40) shown in response to S. epidermidis in invitro conditions [45]
			*Staphylococcus epidermidis* and *Veillonella* [34, 41, 45]	Increase in abundance	- Neonates exposed to *S. epidermidis* can lead to production of antigen-specific CD4 + T cells, which are mostly Treg cells. The delay in the exposure to *S. epidermidis* until adulthood causes inflammatory cytokine-producing effector CD4 + T cells to predominate [34]
					*- S. epidermidis* colonization induces IL17A + CD8 + T cells that can home to the epidermis leading to the enhancement of innate barrier immunity and thereby limits invasion by pathogens [41]
		*Actinobacteria*	*Dermacoccus spp.* [45]	Decrease in abundance	
		Deinococcota	*Deinococcus* [45]	Decrease in abundance	
		Proteobacteria	*Methylobacterium Haemophilus *[45]	Decrease in abundance	
			*Genera Streptococcus,Corynebacteriu*, Cutibacterium [34, 43, 42]		Mycolic acid present in the cell envelope of several members of *Corynebacterium* genus can specifically induce IL-17A + dermal ɣδ T cells [42]
	Gut	*Firmicutes*	*Clostridium difficile* [33, 49],	Increase in abundance	
			*Coprobacillus spp.,*		
			*Peptoniphilus spp.* [54],		
			*Enterococcus spp.* [54, 55]		
			*Fecalibacterium prausnitzii* [59]	Increase in abundance	Chronic progression of atopic dermatitis is associated with dysbiosis of *Fecalibacterium prausnitzii* which causes an impairment of gut epithelial barrier leading to aberrant Th2 type immune responses against allergens in the skin [59]
		*Actinobacteria*	*Eggerthella spp.* [54]	Increase in abundance	
			*Bifidobacterium* [48–50]	Decrease in abundance	*Bifidobacterium longum AH1206* can induce Treg cells and has been shown to be protective against respiratory allergic inflammation [50]
		Proteobacteria	*Escherichia coli* [33]	Increase in abundance	
			*Sutterella spp.* [51, 54, 56]	Decrease in abundance	Endotoxin from Proteobacteria enhances IL-12 production from monocytes and dendritic cells and thereby elicits a Th1 response [56]. Increased risk of atopic eczema is associated with low exposure to the endotoxin [51]
		Fusobacteria	*Fusobacterium spp.* [54]	Decrease in abundance	
		*Bacteroidetes*	*Bacteroides* [52–55]	Decrease in abundance	*- Bacteroides fragilis* suppresses production of inflammatory cytokines TNF-α and IL-23, thereby preventing induction of colitis, in an experimental colitis model [52]
					*- Bacteroides fragilis* elicits mucosal tolerance by mediating conversion of CD41 T cells into IL-10 producing FOXP3 + Tregs as shown in a murine model [53]
**Food allergy**	Gut	Proteobacteria	*Enterobacteriaceae* [77]	Increase in abundance	
			*Citrobacter* [78]	Decrease in abundance (Food allergy)	
			*Haemophilus* [78]	Decrease in abundance (Food sensitization)	
		*Bacteroidetes*	*Bacteroidaceae* [77]	Decrease in abundance	
		*Firmicutes*	*Ruminococcaceae* [77]	Decrease in abundance	
			*Dailister*, *Dorea* and *Clostridium* [73–75, 78]	Decrease in abundance (Food sensitization)	Many members of the Clostridia class possess anti-inflammatory properties via their ability to produce short-chain fatty acids [73, 74] or through alternate mechanisms that leads to reduced IL-12 and IFN-ɣ levels and secretion of IL-10 [75]
			*Oscillospora*, *Lactococcus* and *Dorea* [76, 78]	Decrease in abundance (Food allergy)	*Lactococcus* possess anti-inflammatory properties which involves reduction of TNF-α [76]
**Allergic Rhinitis**		Proteobacteria	*Prevotella*, *Neisseria* and Moraxella species [97]	Increase in abundance	
**Asthma**	Lung	*Firmicutes*	*Selenomonas* species and *Butyrivibrio* species [97]	Increase in abundance	
			*Blautia* and *Lachnospiraceae inserta sedi*s [101]	Increase in abundance in eosinophil-low asthma patients	
			*Aeribacillus, Virgibacillus* and *Caldalkalibacillus* [101]	Increase in abundance in eosinophil-high asthma patients	
		Proteobacteria	*Neisseria* [88, 101],	Increase in abundance in eosinophil-low asthma patients	*Neisseria* species have been shown to have immunomodulatory properties through expression of membrane porins that are established as pathogen-associated molecular pattern recognized by TLR2 on dendritic cells causing elevated levels of IL-6 [88]
			*Halomonas, Sphingomonas, Burkholderia* and *Shewanella* [89, 101]	Increase in abundance in eosinophil-high asthma patients	*Sphingomonas* species have glycolipid antigens that have been reported to induce airway hyperreactivity (AHR) in mice through the activation of NKT cells through an IL-33 dependent manner [89]
			*Haemophilus parainfluenzae* [104]	Increase in abundance in corticosteroid-resistant asthma	
			*Haemophilus* and *Moraxella* species [90, 102, 103]	Decrease in abundance Neutrophilic asthma	*Haemophilus influenzae* and *Moraxella catarrhalis* have been shown to induce high levels of proinflammatory cytokines (IL-8, TNF-α and thymic stromal lymphopoietin) and cause increased airway neutrophil recruitment but with no detectable eosinophil recruitment [90]
		Bacteriodetes	*Prevotella* species [97]	Increase in abundance	
			*Bacteroides* [101]	Increase in abundance in eosinophil-low asthma patients	
		*Actinobacteria*	*Actinomyces* and *Rothia* [101]	Increase in abundance in eosinophil-low asthma patients	
			*Nesterenkoni* [101]	Increase in abundance in eosinophil-high asthma patients	
	Gut	Proteobacteria	*Clostridium neonatalae* [106]	Increase in abundance	
		*Firmicutes*	*Enterococcus and Clostridium* [107]	Increase in abundance	
			*Blautia* and *Ruminococcus* [107]	Increase in abundance in infants at high-risk for asthma	
			*Faecalibacterium* [85, 107] and *Roseburia* [107] *Lachnospira* [85, 106] and *Veillonella* [85]	Decrease in abundance	
		*Actinobacteria*	*Rothia* [85]	Decrease in abundance	

In addition to the role of the skin microbiome, the composition of the gut microbiome was similarly found to be altered in patients with atopic dermatitis who exhibited a low gut microbial diversity which was also associated with disease [48–54]. The composition of the gut microbiome in 1-month-old infants from the KOALA birth cohort study showed that colonization with *Clostridium difficile* led to an increased risk of development of atopic dermatitis and other allergic diseases [33]. Furthermore, atopic dermatitis has been associated with an increased abundance of *Firmicutes*, specifically the *Clostridium difficile*, *Coprobacillus* spp., *Enterococcus* spp., and *Peptoniphilus* spp. and a decreased abundance of Proteobacteria and Bacteriodetes in the intestine [54, 55], potentially due to a lack of exposure to the LPS contained in the cell walls of Proteobacteria, which exerts a protective action through boosting IL-12 production by monocytes and dendritic cells to induce responses [56], which may otherwise be impaired in pediatric atopic dermatitis patients [55]. Bacterial metabolites synthesized by the gut microbiome also play a pivotal role in providing protection against the development of atopic dermatitis [57]. As the intestinal barrier function can be impaired in those patients [58], with impairment often being associated with an enrichment of *Fecalibacterium*, in particular, *Fecalibacterum prausnitzii*, a non-short chain fatty acid (SFCA)-producing bacteria which is especially common in pediatric atopic dermatitis [59] and can lead to an increased inflammation of the gut epithelium in these patients [60]. Alternately, SCFA-producing bacteria can upregulate the expression of tight junction to improve the intestinal barrier function [55]. Moreover, SCFAs like butyrate can regulate the activation and proliferation of colonic Treg cells, which has been shown to be protective in mouse models of disease [57, 61].

## Atopic Food Allergies

Food allergy is a condition that has shown a significant increase in prevalence over the last 20 years [62], now affecting nearly 8% of children and 5% of adults worldwide [63]. The eight most common food allergens in young children are cow’s milk (2.5%), egg (1.3%), peanut (0.8%), wheat and soy (nearly 0.4% each), tree nuts (0.2%), and fish and shellfish (0.1% each) [64]. Food allergies can be broadly classified as resulting from immune pathways that activate effector cells through food-allergen specific IgE or non-IgE-mediated mechanisms, [64, 65]. Oral tolerance is induced under homeostatic conditions and leads to the suppression of immune responses to food-derived foreign antigens encountered in the gastrointestinal tract [66]. The loss of oral tolerance initiates a cascade of immune responses against otherwise innocuous food antigens resulting in food allergy [67]. As at other sites of barrier immunity, Treg cells play a key role in maintenance of tolerance by regulating immune responses to allergens through several mechanisms, including the production of the inhibitory cytokines IL-10 and TGF-β [68], by inhibiting the proliferation of effector T cells, depriving the cells of IL-2 [69] and through the production of granzymes A and B that can cause cytolysis of effector T cells [68]. Several recent findings suggest that the SCFA, butyrate, contributes to the development of oral immune tolerance due to its strong anti-inflammatory effects [70, 71]. Emerging 16S rRNA sequencing–based studies on food allergy and sensitization indicate that gut dysbiosis may precede the development of food allergy (Table 1) [72–76]. The Canadian Healthy Infant Longitudinal Development (CHILD) study indicated that a reduced gut microbial diversity at 3–6 months was associated with an increased tendency for food sensitization at 12 months and showed an increased abundance of Enterobacteriaceae and a decreased abundance of *Bacteroidaceae* and *Ruminococcaceae* [77]. A US pediatric cohort study determined that a lower abundance of *Citrobacter*, *Oscillospora*, *Lactococcus*, and *Dorea* in stool samples collected at 3–6 months of age was associated with food allergy by age three and a relatively lower abundance of *Haemophilus*, *Dailister*, *Dorea*, and *Clostridium* in stool samples of the same age group exhibited food sensitization by age 3 to at least one of the eight major food allergens [78]. Whereas clostridia exhibits a protective effect against sensitization to food allergens through regulation of the innate lymphoid cell function and intestinal cell permeability [55]. Although the gut microbiome changes with time, the most rapid changes occur early in life and are mainly influenced by whether the infants are vaginally delivered or via Cesarean section (C-section) and breast- or formula-fed [79]. In addition, antibodies, such as IgA at the mucosal surface of the intestine, can diffuse across the gut epithelium into the lumen to bind and prevent an inappropriate crossing of intestinal microbiota into the bloodstream [80]. With IgA levels being essential for the maintenance of intestinal homeostasis and the regulation of gut microbiota composition [81], especially during the post-natal period where IgA is transferred to infants through maternal breast milk and plays a vital role in immune and microbial homeostasis. Intriguingly, a recent study by Abdel-Gadir et al. [82] reported a decrease in binding of fecal bacteria to IgA and an increased binding to IgE in infants with food allergy revealing a previously undescribed allergic response to commensals in the intestine of food allergic patients.

## Asthma and Allergic Rhinitis

Asthma is a common allergic inflammatory disease affecting more than 300 million people worldwide [83]. Broadly, asthma can be defined to be of either an atopic or non-atopic phenotype. In atopic asthma, the incidence of asthma symptoms occurs later in childhood or in early teenage years and may be resultant of an underlying genetic predisposition to allergen-sensitivity leading to development of hyper-responsiveness with symptoms mostly persisting into adulthood [84]. Whereas, non-atopic asthma largely develops within the first 2 to 3 years of age and develops as a neutrophil associated, recurring obstruction of the airways that typically resolves by around 13 years of age [84]. Several studies have reported the positive influence of microbial exposure on protection from the development of asthma, with children who are exposed to a highly diverse microbial environment often exhibiting lower rates of asthma and allergic rhinitis (Table 1) [24, 85–90]. For instance, in rural areas, children raised on farms are more likely to be exposed to livestock, as well as an increased likelihood of having consumed unpasteurized milk from farm animals during their early childhood [25, 91]. Such exposures to a microbial environment at a very young age are associated with a relatively lower risk of developing allergic diseases [92]. This is typically referred to as the “farm-effect” on allergic diseases and has been associated with both atopic and non-atopic phenotypes of asthma [92, 93]. Schuijs et al. reported that the ubiquitin-modifying enzyme A20 in the lung epithelium renders the protective effect in children living in farm environment, showing that the loss of A20 enzyme eliminated the protective effect; in addition, a single nucleotide polymorphism in the gene encoding for A20 enzyme was associated with allergy and asthma risk in children raised in a farm environment [94]. It was further shown that farm dust and bacterial LPS modify the communication between epithelial cells and dendritic cells, achieved through the induction of A20 expression [94], providing a possible explanation for the incidence of higher rates of asthma in children from urban areas, as compared to those from rural areas. Allergic rhinitis, also known as hay fever, is an atopic disease characterized by nasal congestion, sneezing, and rhinorrhea [95] and is predominantly caused by allergens such as pollen, dust mites, and animal dander; based on the causative allergen, allergic rhinitis can present as either seasonal or perennial in nature [96]. A recent analysis of the microbial composition in the mucosal airways of children with asthma or allergic rhinitis identified a decrease in certain groups of microbes and linked this microbial dysbiosis with the increased sensitization to allergic disease [97, 98]. Among those microbes found to constitute a healthy lung microbial composition were *Bacteroidetes*, *Actinobacteria*, and *Firmicutes* [83], whereas the phylum Proteobacteria was found to be abundant in asthmatics and was associated with lower levels of asthma control and increased numbers of asthma exacerbations [99, 100]. Other common microbial populations found in the mucosal airways of asthmatic patients include *Prevotella*, *Selenomonas*, *Butyrivibrio*, [97], and *Neisseria* [97, 101]. Some of these species, which includes *Prevotella* and *Neisseria* species, are also found associated with patients with allergic rhinitis [97]. Interestingly, the airway microbiome composition in patients with eosinophilic and neutrophilic asthma was found to be distinct, with the neutrophilic patients exhibiting a reduced diversity and richness in Proteobacteria and in particular, *Haemophilus* and *Moraxella* species [102, 103]. *Haemophilus parainfluenzae* is capable of activating the toll-like receptor (TLR) 4, which in turn leads to the transcription of pro-inflammatory cytokine IL-8 and inhibition of corticosteroid responses [104]. Apart from the airway microbiome, dysbiosis of the gut microbiome and lower gut microbial diversity early in life are also associated with a subsequent increased risk of developing asthma [105]. Data from the CHILD cohort study which examined children who were asthmatic at 4 years of age showed that the gut microbial composition of these children at 3 months of age exhibited a significant decrease in the abundance of the genus *Lachnospira* and an increased abundance of the species *Clostridium neonatalae* [106]. A further study which analyzed the gut microbial composition of infants at risk for asthma, during the first 100 days of their life, showed a decreased abundance of *Lachnospira*, *Veillonella*, and *Faecalibacterium* from the phylum *Firmicute*s and *Rothia* from the phylum *Actinobacteria* [85]. Yet another study showed that children with asthma had a significantly lower abundance of *Faecalibacterium* and *Roseburia*, belonging to the phylum *Firmicutes*, whereas *Enterococcus* and *Clostridium* from the same phylum were enhanced in these children as compared to healthy controls [107].

## Early Life Exposures to Microbes and Immune System Development

### Pre- and Post-Natal Colonization of Barrier Organs by the Microbiota

Until recently, it had been assumed that the prenatal environment was a sterile location, free from microbes. However, several studies published over the last decade have begun to question the “sterile womb” theory and whether prenatal colonization of the developing fetus does in fact occur [108–112]. The concept of a prenatal microbiome remains highly controversial and has been extensively reviewed [113, 114] and debated previously [115, 116]. New findings published in Mishra et al. [117] may present the best evidence for microbial exposure and colonization of fetal organs by the microbiome. The authors showed that fetal organs contained a diverse array of bacterial species and that bacteria isolated from these organs can be grown under in vitro culture conditions. Bacterial structures were additionally visualized by electron microscopy in the 14-week-old fetal gut, with coincident staining for the presence of 16S rRNA by RNA in situ hybridization. Analysis of the T cell compartment revealed the presence of fetal memory T cells that were able to be expanded in the presence of the fetal-isolated bacterial strains. Whether these findings represent the identification of a definitive fetal microbial niche, indicative of a true host-microbe relationship or are merely evidence of persistent or transient colonization, still remains to be determined. However, evidence for the microbial priming of an adaptive immune response during the period of fetal growth has significant implications for the development of the immune system and may play a considerable role in the development of atopic disease susceptibility that has yet to be determined.

The exposure to the microbial environment early in post-natal life plays a significant role in the development of the immune system (see Table 2) [24]. The first few days after birth when neonates get their first major microbial inoculation represents a critical window in their immune system development [9, 118]. During this window, several factors can alter or influence the initial microbial colonization [24]. Among the potential factors, mode of delivery provides an initial strong primary determinant for post-natal colonization of microbial communities and associated barrier functions, playing a major role in the development of the subsequently established microbiome. During vaginal delivery, neonates acquire the major microbial communities from the mother, mainly characterized by an increased abundance of *Bacteroides* and *Parabacteriodes* [119]. In contrast, babies born via C-section receive their first microbial inoculum from other sources, such as skin, saliva, or breast milk [119]. Interestingly, emerging evidence indicates that microbial communities acquired via vertical transmission are capable of adapting quickly to the new environment, and priming the associated immune functions, particularly LPS biosynthesis pathways and two-component systems pathways that are significantly under-presented in neonates born via C-section [120]. LPS, is a membrane component of Gram-negative bacteria and is capable of priming the neonatal immune system by stimulating secretion of pro-inflammatory cytokines at the interface of the earliest gut microbiome, which may result in persistent effects on neonatal physiology, including protective effects towards developing allergies later in life [86, 121]. On the other hand, an early perturbation of the host-commensal priming in neonates born via C-section can lead to defects in the proper education of the immune system [122] and higher propensities to develop chronic diseases later in life [123], with significant increases in the incidence of antibiotic resistant, hospital associated microbes being detected in several studies [124–126].

**Table 2:** Pre- and post-natal microbial colonization of neonatal organs and associated immune functions

***Microbial colonization***	***Type of microbiome***	***Phylum***	***Family/species***
Pre-natal	Skin and gut microbe	*Firmicutes*	*Staphylococcus spp.*, and *Lactobacillus spp.*
Pre-natal	Gut and skin microbe	*Firmicutes*, and *Actinobacteria*	*Enterococcus faecium, Staphylococcus epidermidis, Streptococcus sanguinis*, and *Propionibacterium acnes*
Pre-natal	Oral, gut, skin and vaginal microbe	*Firmicutes*, Tenericutes, Proteobacteria, *Bacteroidetes*, and Fusobacteria	*Prevotella tannerae*, *Neisseria, Ureaplasma*, *Mycoplasma*, and *Escherichia coli*
Pre-natal	Gut and vaginal microbe	*Firmicutes*, *Actinobacteria*, Proteobacteria, and Fusobacteria	*Lactobacillus*, *Gardnerella*, *Haemophilus*, and *Sneathia*
Pre-natal	Vaginal microbe	*Firmicutes*, and *Actinobacteria*	*Lactobacillus* and *Micrococcus luteus*
Post-natal	Gut microbes	Proteobacteria, *Firmicutes*, *Bacteroidetes*, *Actinobacteria*	*E. coli*, *Streptococcus*, *Bacteroides vulgatus*, *Bacteroides*, *Bididobacterium longum*, and *Actinobacteria*
Post-natal	Gut microbes	*Firmicutes*, Proteobacteria	*Enterococcus, Enterobacter* and *Klebsiella*
Post-natal	Skin and oral microbes	Proteobacteria, *Firmicutes*, Proteobacteria	*Enterobacter*, *Haemophilus*, *Staphylococcus saprophyticus*, *S. aureus*, *Streptococcus australis*, *and Veillonella* *Clostridium difficile*, *Granulicatella adiacens*, *Citrobacter spp*., *Enterobacter cloacae*, *Bilophila wadsworthia*
Post-natal	Gut microbes	-	Gram negative commensal
Post-natal	Gut and lung microbes	*Bacteroidetes*	*Bacteroides fragilis*

***Microbial colonization***	***Relative abundance***	***Primary outcome and influence on immune functions***	***Reference***
Pre-natal	Observed in the fetus during the pre-natal period	• Identification of natural bacterial flora in the fetus confirms the microbial exposure and colonization of fetal organs during the pre-natal period • The presence of fetal memory T cells during the pre-natal period indicates microbial priming of an adaptive immune response even before birth	Mishra et al. [117]
Pre-natal	Identified in the umbilical cord blood	• Mother-to-child transmission of healthy microbes during the pre-natal period may boost the neonatal immune functions via the production of short chain fatty acids and may also reduce their susceptibility to acquire undesired pathogens that could be acquired from the hospital environment	Jimenez et al. [108]
Pre-natal	Identified in the placenta	• Placenta harbors a unique microbiome; despite its low abundance, it is metabolically active. However, their influences on the immune function are not discussed	Aagaard et al. [110]
Pre-natal	Identified in placenta and fetal lungs	• Fetal lungs and placenta harbor a microbial signature, which could possibly be transplacentally derived and acquired in utero; however, their influences on immune function are not discussed	Al Alam et al. [111]
Pre-natal	Identified in the fetal intestines	• Fetal intestines harbor a unique microbial signature, which can influence the fetal immune system as early as the first trimester • The presence of *Micrococcus* in the fetal intestines can modulate mucosal immunity and may predispose fetal T cells to develop into regulatory T cells • Fetal intestinal flora dominated by Micrococcus can mount an inflammatory response by inducing tolerogenic APCs and inhibiting IFN-γ production by fetal memory T cells and exhibiting higher PLZF + CD161 + T cell proportion in lamina propria to promote epithelial stem cell function and distinct programs of immune cell recruitment	Rackaityte et al. [112]
Post-natal	Increase in abundance in vaginally delivered neonates	• Natural microbial inoculation during vaginal delivery induces better immune functions in neonates such as lipopolysaccharide (LPS) biosynthesis pathways and two-component systems pathways compared to babies born via a C- section	Wampach et al. [119]
	Increase in abundance in C-section delivered neonates	• LPS can prime the neonatal immune system by stimulating the pro-inflammatory cytokines	Ferretti et al. [120]
	Increase in abundance in neonates delivered via C-section Increase in abundance in formula-fed infants	• Vertical mother-to-infant microbial transmission occurred through multiple sources during pre- and post-natal	
Post-natal	Increase in Gram negative abundance	• Opportunistic pathogens that tend to be found in hospitals are dominated in babies born by C-section. Genome-wide sequencing indicates that these bacteria contain genes responsible for antibiotic resistance and virulence • Babies born by C-section have an increased risk of asthma and obesity later in life	Shao et al. [124] Dominguez-Bello et al. [125] Keag et al. [123]
Post-natal	Ealy gut or lung abundance in fetus	• The gut flora of babies born by C-section is derived from opportunistic colonization and more similar to the skin and oral flora of the mother or the surrounding environment during delivery	Backhed et al. [9]
		• The microbiome of infants delivered by C-section tended to contain 90% higher prevalence of antibiotic resistance genes compared to vaginally delivered infants	Bezirtzoglou et al. [126]
Post-natal		• Early exposure to Gram-negative commensal bacteria strongly influences the host immune system and is inversely associated with asthma and allergic sensitization at school age • Elevated levels of infant home endotoxin and subsequent reduced Th2 cytokine IL-13 production are associated with a reduced risk of allergic sensitization	Prince et al. [118]
Post-natal		• Immune influences induced by the microbiota during early period of the life may be durable, creating a “window of opportunity” for proper or improper immune modulation to occur and resistance (or susceptibility) to disease later in life • Early gut monocolonization of *Bacteroides fragili*s may stimulate a fine balancing of systemic TH1 and TH2 cells possibly due to a single microbial molecule, polysaccharide A (PSA); however, early lung monocolonization of *B. fragilis* can increase susceptibility to asthma • Perturbations of microbial composition during early life may be associated with increased susceptibility to allergy and asthma at 6 years of age	Gensollen et al. [122]

Apart from the mode of delivery, other environmental factors such as feeding with breast milk, staying in a joint family, or farm environment in the first few years of life can increase exposure to vast microbial diversity (Fig. 1), which may result in adequate immune responses against diverse microbial antigens; however, elimination of such microbial exposures either by feeding with formula milk, staying in a nuclear family, or exposures to antibiotics at a young age can promote inflammatory immune responses including those associated with asthma and other allergic diseases [24].

## Pre- and Post-Natal Development of the Adaptive Immune System

During pregnancy, the maternal immune system adopts a complex immunologic mechanism to enable the co-existence and maintenance of an equilibrium between both the maternal and developing fetal immune systems [13, 127]. In order to prevent fetal and placental immune rejection, whilst allowing for the unmatched tissue growth of the fetus as it prepares for adaptation to the external environment and the ensuing microbial colonization that occurs at birth [128]. The thymus becomes a functional organ of T cell development between the 7th and 16th week of gestation [129]. During the 8th week of gestation, early lymphoid progenitors originate from the liver and subsequently migrate into the thymus where they develop into naïve T cells [130]. Circulating T cells are observed around the 10th to 11th week of gestation following the development of a functional thymus [131]. Impaired growth of the fetal thymus has been shown to be related to several complications associated with pregnancy including preeclampsia, a condition which presents with reduced peripheral Treg cells in both the mother and newborn [132] and which in turn is associated with increased risk of allergic diseases development during childhood [133]. The fetal immune system is generally characterized as tolerogenic [134, 135], a feature essential for fetal survival [136]. During the gestational period, a substantial number of maternal cells cross the placenta to reside in the fetal lymph node which provokes the development of CD4^+^CD25^hi^FOXP3^+^ Treg cells. Various cytokines, hormones, and bacterial products including SCFAs and lipopolysaccharides are also involved in transplacental immune regulation [132, 137]. Fetal Treg cells suppress the proliferation and cytokine secretion of other potentially self-reactive T cells [134]. Human cord blood and infant blood are both characterized by a predominance of Th2 and Treg cells, as compared to Th1 or Th17 cells, which are more restricted in early life [134]. In fact, the Th2 and Treg phenotype bias in fetal tissues develops as early as the second trimester of pregnancy as identified from fetal spleen and lymph nodes [135]. A recent study described the differential expression of arginase-2 between fetal and adult splenic dendritic cells resulting in the fetal dendritic cells to favor the induction of Treg and Th2 cells over Th1 cells [138]. Human cord blood studies have also shown that neonates have negligible amounts or complete absence of Th17 cells [139] that play significant role in developing immunity against fungal and bacterial infections at epithelial barriers [140]. Tregs and Th17 cells have reciprocal development pathways in the absence of the pro-inflammatory cytokines IL-6, IL-1β, and IL-23 wherein Foxp3 dominates RORγt function and prevents Th17 development. Thus, neonatal immunity is typically considered to exhibit an anti-inflammatory profile as the Th2 and Treg phenotype bias in early life impairs Th1 and Th17 immune responses [141] but may instead lead to a bias towards the development of an atopic phenotype.

## Mechanisms of Microbial Effects on Adaptive Immunity

### Immuno-Microbiome Interactions Alter T Cell Differentiation in Allergic Diseases

The human body is constantly exposed to an external microbial environment comprised of a wide variety microbial species able to colonize the barrier organs of the human body forming symbiotic, commensal, or pathogenic relationships with the host [142, 143]. During development the immune system co-evolves in the presence of these microbes and plays an important role in maintaining the homeostatic balance between tolerance and allergy, through regulation of host-microbiome interactions. Recently, the influence of the gut microbiota in the development of allergic disease has become widely studied, revealing that during early developmental stages, the immune system becomes tolerized to commensal bacteria, through the induction of a corresponding complement of IgA antibodies and regulatory T cells, enabling the commensal bacteria present to be maintained in the gut over time [35]. Interestingly, it has been shown that compositionally and functionally distinct gut microbiota exist at different stages of neonatal development and exert differential influences on the immune system [32]. During development, gut microbial dysbiosis may occur which can alter the homeostatic balance of the immune system leading to a cascade of events that results in an allergic outcome [78]. Animal-based studies have identified the presence of at least two distinct types of Tregs in the gastrointestinal tract — thymically-derived Treg (tTreg) and peripherally induced Treg (pTreg), the latter being predominantly enriched in the intestines and largely responsible for maintaining tolerance to food and microbiota-derived antigens at mucosal surfaces [144], with the composition of the gut microbiome influencing the differentiation of pTregs in an antigen-specific manner [145]. Germ-free animal studies indicate that pTregs fail to differentiate in the gastrointestinal tract of mice lacking a diverse microbiota, whereas a normal tTreg compartment was maintained in these animals [144]. Furthermore, it has been reported that dietary antigens can induce the differentiation of pTregs, with this population being distinct from the microbiome-induced pTregs, in that they co-express both Foxp3 and GATA3, have a limited life-span, and can repress the strong immunity to ingested protein antigens [146]. Although most of these studies have been conducted in animal models, recent clinical studies on human subjects identified that Tregs are not functionally impaired in individuals with allergies, but there is a striking increase in the proportion of reactive Th2 cells in these individuals [147], indicating that antigen escape from Treg control was the dominant factor associated with the loss of tolerance observed in allergic individuals.

### Th2/Treg Axis and the Microbiome

Under homeostatic conditions, the adaptive immune system normally develops a system of tolerance to harmless environmental antigens largely mediated by members of the T cell lineage, particularly Treg cells [147]. Tregs play a dual role in the intestine, by maintaining immune tolerance to dietary antigens [148] and limiting the potentially damaging immune responses that can be generated against environmental pathogens [149]. However, when there is a failure in generation of tolerance by Treg cells against common food and aero-antigens, it can result in the differentiation of allergen-specific cells of the Th2 lineage and the associated production of atopy-inducing IgE [150]. Along with a cascade of downstream events triggered by production of cytokines including IL-4, IL-5, IL-9, and IL-13, ultimately leading to the recruitment and activation of a raft of innate immune cells including basophils, eosinophils, ILC2’s, and M2 macrophages, which together cause the pathology associated with allergic disease [151]. A subset of human memory Th2 cells, allergen specific Th2 cells have been recently reported to be confined only to atopic individuals, referred to as Th2A cells. These Th2A cells are terminally differentiated and co-express CRTh2, CD49d, and CD161 and are thought to be functionally distinct from conventional Th2 cells [152], as they may be more readily activated by perturbations in mucosal barrier function and activation by the “alarmin” cytokines TSLP, IL-25, and IL-33 [153]. Treg cells, on the other hand, produce the anti-inflammatory cytokines, TGFβ, and IL-10, which among other regulatory mechanisms are key in protecting the host from excessive inflammatory immune responses [154]. Hence, a balance in the Th2/Treg axis is essential to protect against the development of allergic disease [155]. Studies in animal and human systems have begun to elucidate several potential mechanisms that tie the microbiome and microbial diversity to alterations in the regulation of Th2 and Treg differentiation leading to a cellular and molecular rationale for those observations made by the hygiene hypothesis. Intriguingly, studies of germ-free mice lacking a microbiome found that a complete microbial dysbiosis led to the establishment of a default Th2 biased immune environment [156, 157], reminiscent of that observed during neonatal and early post-natal life [134, 135, 138]. Indicating that the introduction of commensal bacteria at an early stage may be critical for ensuring normal cellular maturation and recruitment in order to control allergic inflammation. Studies into the relative roles of tTreg and pTreg cells revealed that animals deficient for pTreg had altered ratios of *Firmicutes* to *Bacteroidetes*, which was associated with the spontaneous induction of Th2-associated mucosal inflammation and lung pathology [158]. Revealing that pTreg are essential for regulation of the homoestatically controlled microbial community in the gut, through exerting control over Th2 mucosal inflammation and regulating the induction of Th17 differentiation which enables B cell production of IgA and the establishment of a “mucosal firewall” [159]. A key component of this microbial regulation of type 2 inflammation was shown to be induced through the induction of intestinal RORγt + pTregs, which modulate the differentiation of Th2 cells in a CTLA-4-dependent manner by regulating the co-activator functions of stimulatory DC [160], thereby balancing immune responses at the mucosal surface through the induction of Th17 and Treg cells [145].

### Effects of Microbial Products on T Cell Immune Responses

**Fig. 2:**
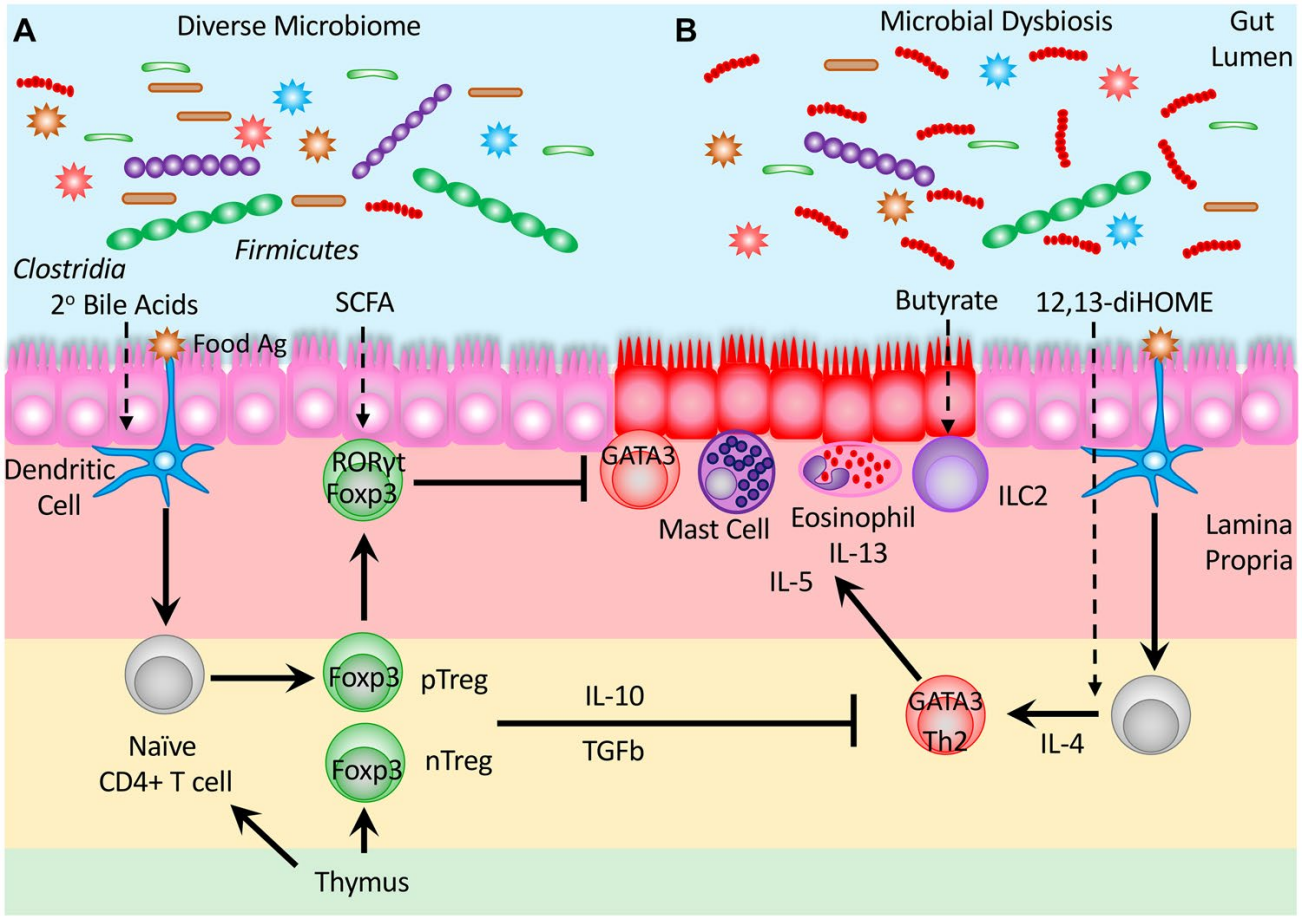
Microbial Product Signaling Influences T Cell Immune Responses in Homeostasis and Dysbiosis. **A** A diverse microbiome promotes homeostatic maintenance and the induction of immune tolerance. Crosstalk between the microbiome and the immune system is mediated by microbial products that promote the differentiation of pTreg and the upregulation of RORγt in response to stimulation with food derived antigens. **B** Microbial dysbiosis results in the production of microbial products that promote inflammatory responses and modulate Th2 differentiation in response to the presentation of Ag’s and stimulate ILC2 recruitment, resulting in type 2 inflammation in affected tissue following the recruitment of innate cell mediators of disease including mast cells and eosinophils

Multiple studies have now shown that specific products of metabolism synthesized by the component species of the microbiome are able to exert effects on the differentiation and function of CD4 + T cells [161], providing a mechanistic rationale that directly links microbial dysbiosis with the alterations in immune function that lead to allergic disease (Table 3). Some of the earliest studies linking microbial metabolites with alterations in immune function focused on the effects of antibiotics on disrupting microbial homeostasis, as the use of antibiotics at an early age is a known risk factor associated with increased risk for atopy [161]. Murine models have revealed that along with the induction of a microbial dysbiosis, antibiotic treatment also caused a concomitant decrease in protective gut Treg cells and the induction of inflammation. Intriguingly, this decrease in Treg cells and the associated inflammatory response could be inhibited by treatment with SCFA [162] (Fig. 2). Under homeostatic conditions, SCFA are produced by the microbial conversion of dietary fiber by anaerobic fermentation [163], especially by those microbial taxa associated with protection, including *Clostridia* and *Firmicutes*.

**Table 3:** Effects of microbial products on T cell immune responses

***Metabolic product***	***Microbial*** ***source***	***CD4 + T cell effects***	***Mechanism***	***Reference***
Short Chain Fatty Acids	*Clostridia* *Firmicutes*	Enhanced generation of intestinal pTreg	Increased levels of TGFβ through activation of enhanced secretion by epithelial cells	[57, 61, 145, 160, 163, 164]
Butyrate	*Clostridia Firmicutes*	Induction of Treg differentiation	Enhanced histone acetylation at promoter and CNS1 & 3 sequence regions of the Foxp3 locus Upregulates RORγt + expression in Treg	[61] [160]
Propionate	*Clostridia Firmicutes*	Enhances Treg differentiation	Binds to CD4 + T cell receptor GPR43, to reduce histone deacetylase activity (HDAC6 and HDAC9)	[163]
Bile acids	Commensal Bacteria	Induction of colonic RORγt-expressing Treg cells	Signals via farnesoid X receptor (FXR) to induce Tregs in a CNS1 dependent manner	[167]
Microbial polysaccharides	*B. bifidum* *B. fragilis*	Induction of Treg differentiation	Acts via Toll like receptor-2 signaling pathway in peripheral dendritic cells	[170, 171]
Folate (Vitamin B9)	*Bifidobacterium, Lactobacillus*	Promotes survival and maintenance of Treg	Enhanced expression of anti-apoptotic Bcl2 in Treg cells	[172]
Niacin (Vitamin B3)	Commensal bacteria	Enhances Treg differentiation and IL-10 production	Activation of Gpr109a niacin receptor to induce IL-10 expression in antigen presenting cells	[173]
Indole-3-lactic acid (IDO)	*L. reuteri* *B. infantis*	Silence both Th2 and Th17 cells effector function	Signaling through AhR expressed on CD4 + to upregulate Galectin-1	[177, 178]
12,13-diHOME	*E. faecalis* *B. bifidum*	Increased Th2 differentiation and IL-4 production	Acts directly on CD4 + T cells and exerts effects on PPARγ signaling in dendritic cells	[168, 169]

Generation of protective intestinal pTreg can be driven by SCFA including butyrate [145] and propionate [160] in conjunction with bacterial antigens [164]. Generation of the type 2 response suppressing RORγt + Tregs was found to be largely dependent on the presence of SCFA butyrate [61, 165], providing an intriguing molecular post-biotic link between the microbiome and regulation of inflammation. Butyrate has also been shown to play a distinct role in the regulation of type 2 innate lymphoid cells (ILC2s), which are associated with the induction and exacerbation of asthma. Here, butyrate was found to alter HDAC2 activity inhibiting GATA3 expression and ILC2 proliferation [166]. It should be noted that whilst SCFAs contribute to mucosal immune homeostasis, excessive or suboptimal levels of SCFAs have been reported to be associated with inflammation and cancer [167]. The microbiome has also been observed to modify a range of host-derived molecules into steroidal compounds with de novo biological activities and immune functionality [160].

Bile acids which usually aid in the emulsification of dietary fats can also undergo bacterial transformation in the colon into the secondary bile acid 3β-hydroxydeoxycholic acid, which in turn can modulate DC function and facilitate the differentiation of, and increase in the levels of RORγt + pTregs present in the intestinal mucosa [168] that are key for regulating the onset of spontaneous type 2 inflammation [169]. The activation of DC by microbial polysaccharides acting through Toll like receptor-2 (TLR2) signaling has also been demonstrated to play an anti-inflammatory role in the intestine via the induction of Treg and an increase in local IL-10 production [170, 171]. The importance of the B vitamins folate (B9) [172] and niacin (B3) [173] in the maintenance and regulation of function in the intestinal Treg compartment has previously been described, along with studies linking folate with both protective effects against asthma [174] and increased rates of food allergy [175]. Hence, future studies are needed to confirm the direct mechanistic action of these metabolites in the context of allergic disease. Colonization of the gut with *L. reuteri* was previously shown to induce protective Treg cells in an allergic airway mouse model [176]. More recently, studies have indicated that a tryptophan metabolite Indole-3-lactic acid (IDO) produced by both *L.reuteri* [177] and *B. infantis* were able to silence both Th2 and Th17 cells through upregulation of galectin-1, demonstrated in human studies [178].

In a longitudinal study focused on multisensitised atopic children, it was determined that alterations in the gut microbiome and in metabolic activity were evident as early as 3 months of age, during which a distinct fecal microbiome and metabolome were present in those children who went on to develop atopy at 3 years of age. Fecal metabolite extracts isolated from these subjects induced an increase in the relative proportion of IL-4 expressing Th2 cells, whilst also exhibiting Treg suppressive capabilities [168]. Subsequently, one of the compounds identified in this study, 12,13-diHOME a linoleic acid metabolite, was shown to exacerbate lung inflammation in mice and elevated levels of the compound were detectable in neonatal children at 1 month of age, who later went on to develop atopy by age two [169], providing further evidence of the importance of the gut microbiota and the microbial products that it produces in conditioning of the immune system at an early time in life.

## Microbial Dysbiosis and the Development of Allergic Diseases

### The Hygiene Hypothesis

**Fig. 3:**
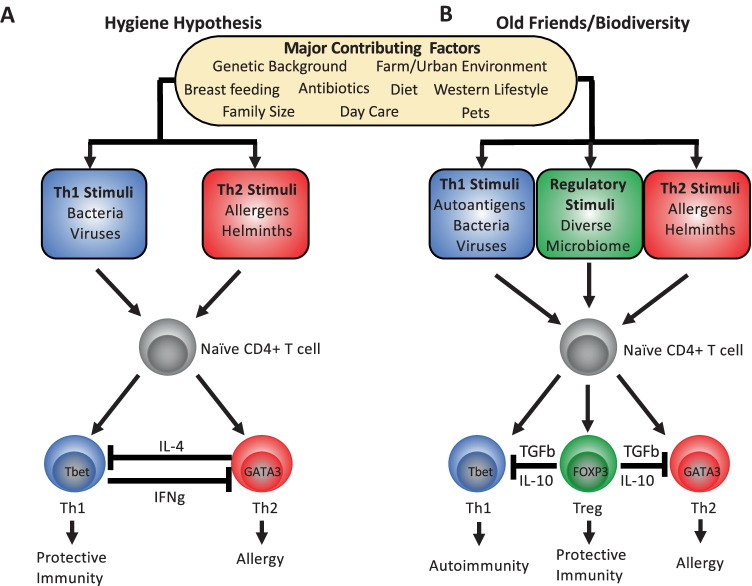
Cellular Basis for Hygiene Hypothesis and Old Friends/Biodiversity models. **A** The original hygiene hypothesis focused on an absence of a sufficient viral/bacterial pathogenic burden to educate the immune system in childhood, allowing for the induction of an aberrant Th2 response. Mechanistically this was seen due an imbalance in the reciprocal regulatory relationship that exists between Th1 and Th2 responses mediated by IL-4 and IFN-ɣ. **B** Old friends/biodiversity hypotheses’ additionally factors in the presence of regulatory mechanisms that are essential for control of both autoimmunity and allergic responses and are associated with the presence of a diverse microbiome. Here, the lack of sufficient regulatory responses accounts for the parallel rise in both autoimmune disease (Th1) and allergy (Th2) seen in recent history

The “hygiene hypothesis” concept dates back to a longitudinal study published by Strachan in 1989 [179] and was initially put forward as an explanation for the emergence of hay fever as a “post-industrial revolution epidemic.” The data published in Strachan’s study established a correlation between hay fever and house size and noted that as the number of older children in the house rose, the incidence of hay fever in younger siblings decreased, leading to the hypothesis that allergic diseases may be associated with a lack of early childhood exposure to infectious disease, spread by unhygienic interactions with older children. Hence, a general decrease in family size and increase in personal cleanliness, along with a concomitant decrease in exposure to Th1 skewing infections during early childhood caused the increased rates of atopic disease [180]. This initial correlation between rates of early childhood infection with the incidence of atopy was subsequently assessed more directly in multiple studies. An analysis of herpes simplex virus infection rates during the first 3 years of life indicated that infection was protective against asthma [181] and a Brazilian study indicated that higher infectious burdens during early life, as measured by plasma Ig levels for exposure to multiple pathogens including herpes simplex virus, Epstein-Barr virus, *Toxoplasma gondii and Helicobacter pylori*, correlated with lower levels of atopy [182]. Additional studies of tuberculosis infection [180], varicella infection [183], and BCG vaccination [183] also found an association with lower rates of atopic disease. These findings indicate that early life exposures to bacterial and viral infections and the production of a strong Th1 response were important in promoting a protective environmental milieu that skews the immune system away from the development of an atopic disease inducing Th2-biased system (Fig. 3).

 However, several arguments against this Th1-Th2 cytokine shift paradigm have emerged. Large-scale and longitudinal cohort studies from the UK [184], Denmark [185], and Finland [186] concluded that after adjustment for clinically apparent infectious diseases, a protective effect of number of siblings, day care, pet ownership, and farm residence was instead responsible for the decreased odds ratios observed (Table 4). Additionally, as atopic disease rates have increased, a concomitant rise in cases of autoimmune diseases has also been observed in children. Including diabetes mellitus (T1D), Crohn’s disease, and multiple sclerosis [187]. As these diseases are largely dominated by Th1 and/or Th17 responses [188], it seems unlikely that the Th1-Th2 cytokine shift paradigm is robust enough to explain the increased development of both sets of disease. Especially in light of findings that indicate there may in fact be an association between the incidence of allergic and autoimmune diseases [189–191]. Furthermore, a large-scale retrospective cohort study (1990–2018) conducted in the UK concluded that the long-term risks of autoimmune disorders are significantly higher in patients with allergic diseases [192]. A recent meta-analysis of the commonalities in 290 genetic loci previously associated with 16 autoimmune diseases, found a significant enrichment of multiple loci also associated with allergy, suggesting that a further investigation of shared mechanisms may help in understanding the complex relationship between these disease syndromes [193]. Contrary to what might have been predicted by the Th1-Th2 cytokine shift paradigm, infections with helminths result in a Th2-polarized immune response including production of IL-4, IL-5, IL-13, eosinophilia, and high serum titers of IgE, all hallmarks of allergic disease. Whilst at the same time, helminthic infections have largely been associated with inducing protective effects on the development of atopic disease as well as naturally occurring infection with *Trichuris trichiura*, *Enterobius vermicularis*, and *Schistosoma mansoni*, which have all been shown to exhibit a protective effect [194], especially when exposure was found to occur during in utero development [195] or early in life [196, 197]. However, the protective effects of helminths do not appear to be universal, for example, infection with *Ascaris lumbricoides* or *S. mansoni* has also been associated with higher asthma rates in certain populations [198–200].

**Table 4:** Summary of studies showing associations between environmental factors linked to the hygiene hypothesis and atopic disease

***Environmental factor***	***Effect on atopic disease risk***	***Cellular response***	***Inflammatory response***	***Antibody response***	***Reference***
**Decreased family size**	↑ Allergic rhinitis	–	–	–	Strachan [179]
	↑ Allergic rhinitis	–	–	–	Genuniet et al. [26]
	↑ Atopic dermatitis	–	–	–	Benn et al. [185]
**Infectious disease**					
- Tuberculosis	↓ Asthma	↑ Th1	↑IFNγ ↓ IL4, IL5	↓ IgE	Shirakawa et al. [180]
- HSV	↓ Asthma	–	–	↓ IgE	Illi et al. [181]
- HSV, EBV, *T. gondi* & *H. pylori*,	↓ Atopy	↓ Th2 ↓ Treg	↓ IL5 IL13 IL10	↓ IgE	Figueiredo et al. [182]
- Varicella	↓ Atopic dermatitis	–	–	–	Silverberg et al. [183]
- Infection history (30 Infections)	No associated risk	–	–	–	Bremner et al. [184]
- Infection history (< 6 months)	No associated risk	–	–	–	Benn et al. [185]
- Infection history (Respiratory/Enteric)	No associated risk	–	–	–	Dunder et al. [186]
**Parasitic Infection**		–	–		
- *T. trichiura*	↓ Skin reactivity	–	–	–	Rodrigues et al. [234]
- *A. lumbricoides*	↓ Atopic dermatitis	–	–	↑ IgE IgG4	Cooper et al. [196]
- *S. mansoni*	↑ Asthma	–	–	–	Leonardi-Bee et al. [198]
- *N. americanus*	↓ Atopic dermatitis	–	–	↑ IgE sIgE	Araujo et al. [200]
- Maternal helminth infection	↓ Asthma	–	–	–	Leonardi-Bee et al. [198]
- Anthelminthic usage (Ivermectin)	↓ Eczema	–	–	–	Elliot et al. [195]
	↑ Eczema	–	–	–	Endara et al. [197]
**Farm residence**	↓ Atopic dermatitis	–	–	–	Benn et al. [185]
	↓ Allergic rhinitis	–	–	–	Genuneit et al. [26]
	↓ Atopic dermatitis	↑ Treg ↓ Th2	↓ IL4 IL5 IL13	↓ IgE	von Mutius and Vercelli [92]
			↑ IFNγ IL10 Tgfβ		
**Pet ownership**	↓ Atopic dermatitis	–	–	–	Benn et al. [185]
**Day care**	↓ Atopic dermatitis	–	–	–	Benn et al. [185]
	No associated risk	–	–	–	Dunder et al. [186]
**Breast milk vs. formula feeding**	↓Asthma ↓ eczema	–	–	–	Heine [10]
	↓Asthma ↓ eczema	–	–	–	Oddy [11]
	↓ Food allergy	–	–	–	Wang et al. [12]
**Antibiotic usage during infancy**	↑ Asthma	–	–	–	Pitter et al. [17]
	↑ Asthma	–	–	–	Slob et al. [16]
	↑ Asthma	–	–	–	Yassour et al. [15]
**Delivery by Cesarean birth**	↑ Asthma	–	–	–	Lin et al. [19]
	No associated risk	–	–	–	Juhn et al. [20]

Although, the “hygiene hypothesis” has been widely accepted by both the scientific community and the general public, it is not without its limitations. Care needs to be taken in terms of interpreting the message when associating hygiene with the pathogenesis of atopic disease [201]. The hygiene hypothesis has however been pivotal in framing the idea that the immune system is still relatively naïve at birth and whilst the adaptive immune system has gone through an internal developmental process to largely limit the number of cells present capable of mounting a response against self, it still needs to be “fed” with information about how to interpret antigens present in the local environmental. Recent studies indicate that this process may largely occur within the first few months following birth through constant contact with microbes from the external environment, as well as via transfer from other humans, especially from close maternal contact. When such microbial exposure is inadequate, the mechanisms regulating the immune system can fail, resulting in autoimmune and allergic diseases [202].

## Current Perspectives

The “old friends” hypothesis, put forward by Rook in 2003 [203], and the “biodiversity hypothesis” of allergy proposed by Haahtela [204] have subsequently emerged, both of which postulate a similar theme that the emergence of allergic reactions is an outcome of a lack of symbiotic relationships with parasites, viruses, and bacteria which have been beneficial for evolution in the past [205]. These hypotheses are also sometimes referred to as “Western lifestyle hypotheses.” The western lifestyle is being generally characterized by minimal or no physical activity among children with most of the time being spent indoors leading to obesity among children and also exposure to increased allergenic burdens, especially those found indoors, including house dust mites (HDMs) [205], thereby leading to a massive shift in the human disease spectrum from infectious diseases to allergies. In addition, excessive use of antibiotics, with an average of approximately 2.5 antibiotic doses per 100 people/day in Western countries [206], and increased use of sanitation technologies have resulted in elimination of certain eukaryotic symbionts, including helminths and protists from the human gut ecosystems [207]. Taken together, these environmental factors associated with westernized lifestyle trigger dysbiosis by affecting intestinal epithelial cell metabolism, sequestering nutrient sources [208], and creating a favorable environment for facultative anaerobes-such as pathogenic *Escherichia coli* and *Salmonella* [209, 210] at the expense of symbiotic flora such as *Bacteroides*, *Prevotella*, *Desulfovibrio*, and *Lactobacillus* [211, 212]. The microbial dysbiosis induced by a westernized diet or lifestyle may also result in a leaky mucosa and reduced intestinal production of short chain fatty acids (SCFAs) [213]. A prolonged microbial dysbiosis may lead to leakage of pathogen-associated molecular patterns (PAMPs), including LPS, in the blood, and trigger low-grade inflammation or allergy [213, 214], ultimately resulting in a change in the lung, gut, and skin microbiomes causing microbial dysbiosis which leads to a sharp decrease in infectious diseases and a higher prevalence of allergies (Fig. 3).

## “Unhygienic Therapies” for Atopic Diseases

As the “hygiene hypothesis” postulates that a lack of microbial interactions in early life leads to an increased risk of atopic disease, the reverse correlate of this implies that the introduction of microbes or use of “unhygienic therapies” may be beneficial in restoring the missing constituents of microbial communities for either treatment or prevention of disease (Tables 5, 6, and 7). The treatment of immunocompromised patients suffering from recurrent *Clostridium difficile* infections has demonstrated the amazing utility of fecal microbiota transplants, where fecal transplant has revolutionized the management of disease leading to a cure rate of 90% after treatment [215]. Although there have been limited studies to date, looking at the use of fecal transplant for treatment of allergic disease in humans [216], a recent study has demonstrated that the transfer of fecal material from healthy infants into a germ-free mouse model was protective against an anaphylactic response to cow milk allergens, whereas colonization of the murine gut with the microbiome from cow’s milk allergic infants was unable to confer protection [217]. Additionally, the transfer of the skin microbiome has become a recent area of interest with the topical microbiome transplantation of *R. mucosa* demonstrating efficacy for treatment of both pediatric and adult atopic dermatitis [218] and more recently, the use of bacteriotherapy was shown to decrease the incidence of *S. aureus* in AD patients [219]. Whilst these studies do provide hope for use of microbial treatment for allergic disease, a large number of studies have examined the possibility of using probiotic supplementation to either prevent or treat disease (Table 1). The majority of probiotic studies into food allergy and atopic dermatitis have investigated administration of either a single or the combination of a limited number of bacterial species and have been shown to have limited utility in either preventing or treating allergic disease [220–228]. Probiotic intervention strategies have also been widely trialed as a potential prophylactic therapy for asthma both during pregnancy [229] or during infancy [230–232]. However, the meta-analysis of these studies has not revealed any substantial protective benefits which could be derived from the current probiotic therapies trialed.

**Table 5:** Therapeutic approaches to treatment of allergic disease with microbes and microbial products: studies investigating the utility of probiotic therapy on allergic disease outcomes

***Allergic condition***	***Microbial treatment***	***Study outline***	***Follow up period***
**Peanut Allergy**	*Lactobacillus rhamnosus*	62 pediatric subjects (ages 1–10) double-blind, placebo-controlled randomized trial of the probiotic *Lactobacillus rhamnosus* and peanut oral immunotherapy (PPOIT). Analyzed unresponsiveness at 2 and 5 wks. Following treatment. (NB: No OIT without PP arm was included.)	2 years
Cow milk allergy	*Lactobacillus casei* and *Bifidobacterium lactis*	119 infants with CMA took part in a double-blind, randomized, placebo-controlled trial. Received CRL431 and Bb-12 supplement added to a standard treatment of extensively hydrolyzed formula for 12 months	6 months and 12 months
Cow Milk Allergy	*Lactobacillus* GG	80 infants with CMA took part in a double-blind, randomized, placebo-controlled trial. Group 1 received extensively hydrolyzed casein formula (EHCF), group 2 received ECHF + *Lactobacillus* GG for 12 months	18 months
Cow Milk Allergy (Non-IgE)	*Bifidobacterium breve*	A total of 35 (test) and 36 (control) subjects were randomized and received test or control formula for 8 weeks. Test formula was a hypoallergenic, nutritionally complete AAF including a prebiotic blend of fructo-oligosaccharides and the probiotic strain Bifidobacterium breve M-16 V. Control formula was AAF without symbiotic	8 weeks
Atopic dermatitis	*Lactobacillus rhamnosus* or *Bifidobacterium animalis*	425 infants from a high-risk birth cohort participated. Maternal supplementation from 35 weeks gestation until 6 months if breastfeeding and infant supplementation until 2 years with either *Lactobacillus rhamnosus* or *Bifidobacterium animalis*	4 years
Atopic Dermatitis	*Lactobacillus* GG	250 pregnant women carrying infants at high risk of allergic disease were recruited to a randomized controlled trial of probiotic supplementation (*Lactobacillus* GG) from 36 weeks gestation until delivery. Infants were then assessed during their first year for eczema or allergic sensitization	1 year
Atopic Dermatitis	*Roseomonas mucosa*	10 adult and 5 pediatric patients were enrolled in an open-label phase I/II safety and activity trial for *R. mucosa* transplantation. Patients self-administered topical bacteria over a 4 or 6 wk. period, results were assessed at 12 wk	4 months
Atopic Dermatitis	*Lactobacillus GG*	132 high risk pregnant women took part in a double-blind, randomized placebo-controlled trial. Lactobacillus GG was administered prenatally to mothers and postnatally for 6 months to their infants	2 years
Atopic Dermatitis	*Lactobacillus rhamnosus GG, L. acidophilus La-5 and Bifidobacterium animalis subsp. lactis Bb-12*	415 pregnant women participated in a randomized, double-blind trial of children from a nonelected maternal population. Women received probiotic milk or placebo from 36 weeks of gestation to 3 months postnatally during breastfeeding	2 years
Atopic dermatitis rhino-conjunctivitis	*L. rhamnosus LC705, Bifidobacterium breve Bb99 and Propionibacterium freudenreichii ssp. shermanii JS*	1223 mothers were recruited during pregnancy and randomized at 35 weeks of gestation to the probiotic or placebo group. Mothers twice daily received one capsule containing a mixture of probiotics or placebo. Their infants were given the same capsules opened and mixed with galacto-oligosaccharides syrup (prebiotics) once daily from birth, continuing to 6 months after birth	5 years and 10 years

***Allergic condition***	***Study outcomes***	***Reference***
**Peanut Allergy**	Possible sustained unresponsiveness was achieved in 82.1% receiving PPOIT and 3.6% receiving placebo (*P* < .001). PPOIT was associated with reduced peanut skin prick test responses and peanut-specific IgE levels and increased peanut-specific IgG4 levels	Tang et al. [220]
Cow milk allergy	No significant difference in tolerance to cow milk at 6 and 12 months was found with: 77% tolerance in the probiotics group compared to 81% in the placebo group	Hol et al. [221]
Cow Milk Allergy	Infants in group 2 had a higher probability of acquiring tolerance at 6 and 12 months compared with subjects in group 1. In infants with IgE mediated CMA SPT responses decreased in both groups after 6 and 12 months, although the difference was not significant	Berni Canani et al. [222]
Cow Milk Allergy (Non-IgE)	At week 8 participants showed statistically significant differences (*P* < 0.001) between test and control groups in the fecal composition of Bifidobacteria and ER/CC. Analyses of clinical outcomes revealed no statistically significant differences were observed at week 8	Candy et al. [223]
Atopic dermatitis	Prevalence of eczema by 4 years and prevalence of rhinoconjunctivitis at 4 years were significantly reduced in the children taking *L. rhamnosus*. There were also non-significant reductions in the cumulative prevalence of wheeze and atopic sensitization. *B. animalis* did not affect the prevalence of any outcome	Wickens et al. [224]
Atopic Dermatitis	Prenatal probiotic treatment was not associated with any change in cord blood immune markers. Prenatal treatment with *Lactobacillus* GG was not sufficient for preventing eczema	Boyle et al. [225]
Atopic Dermatitis	*R. mucosa* treatment was associated with a significant decrease in measures of disease severity, including pruritis, topical steroid requirement, and S. aureus burden	Myles et al. [218]
Atopic Dermatitis	The prevalence of eczema in the treated group was significantly reduced at 2 years of age compared to placebo, (15/64 [23%] vs 31/68 [46%]). Lactobacillus GG was effective in prevention of early atopic disease in children at high risk	Kalliomaki et al. [226]
Atopic Dermatitis	Probiotics administered to nonelected mothers reduced the cumulative incidence of AD, with a reduced OR of 0.51 at 2 years of age, but had no effect on atopic sensitization	Dotterud et al. [227]
Atopic dermatitis rhino-conjunctivitis	Perinatal probiotics decreased eczema up to 10 years of age (35.2% vs 41.7%, adjusted *OR*: 0.74; 95% *CI*: 0.55–1.00; *P* < .05), but at 5–10 years, allergic rhino-conjunctivitis was increased (33.2% vs 26.3%, *OR*: 1.39; 95% *CI*: 1.03–1.89; *P* = .03)	Peldan et al. [228]

**Table 6:** Therapeutic approaches to treatment of allergic disease with microbes and microbial products**:** meta-analyses of microbial treatment strategies for allergic diseases

***Allergic condition***	***Meta-analysis summary***	***Conclusions***	***Recommendations***	***Reference***
Asthma, wheeze	20 eligible trials identified including 4866 children. Heterogeneous in the type and duration of probiotic supplementation, and duration of follow-up. Five trials conducted follow-up beyond participants' age of 6 years with a median of 24 months, none were powered to detect asthma as the primary outcome	No evidence to support a protective association between perinatal use of probiotics and doctor diagnosed asthma or childhood wheeze. Randomized controlled trials to date have not yielded sufficient evidence to recommend probiotics for the primary prevention of these disorders	Extended follow-up of existing trials, along with further clinical and basic research, are needed to accurately define the role of probiotics in the prevention of childhood asthma	Azad et al. [229]
Atopic dermatitis, asthma, allergic rhinitis, wheeze	17 eligible studies identified. Heterogenous in type, duration and conditions assessed. Reporting data from 4755 children (2381 in the probiotic group and 2374 in the control group), were included in the meta-analysis	Infants treated with probiotics had a significantly lower risk ratio for atopic dermatitis compared to controls especially those administered a mixture of probiotic supplements. No significant difference in terms of prevention of asthma, wheezing or rhino conjunctivitis was determined	Results of the meta-analysis show probiotic supplementation can aid in preventing infantile eczema, suggesting a new potential indication for probiotic use in pregnancy and infancy	Zuccotti et al. [230]
Asthma, wheeze	19 eligible studies were identified. Randomized trials involving reporting data from 5157 children fulfilled the inclusion criteria. Trials were heterogenous in type and duration of probiotic supplementation administered	There was no significant association of probiotics with risk of asthma or wheeze compared with placebo. Subgroup analysis by asthma risk indicated that probiotics significantly reduced wheeze incidence among infants with coincident atopic disease	Probiotic supplementation compared with placebo groups did not show an association with a lower risk of asthma in infants. The findings reported here do not support a recommendation of probiotics use in the prevention of asthma in infants	Wei et al. [231]
Asthma, wheeze, eczema	Assessed 21 randomized controlled trials via systematic review and metanalysis where appropriate	Assessed evidence did not indicate that probiotic supplementation significantly reduces the risk of children developing allergy. Considering all critical outcomes, the WAO guideline panel determined that there is a likely net benefit from using probiotics resulting primarily from prevention of eczema	WAO guideline panel suggests: a) using probiotics in pregnant women at high risk for having an allergic child; b) using probiotics in women who breastfeed infants at high risk of developing allergy; and c) using probiotics in infants at high risk of developing allergy. All recommendations are conditional and supported by very low-quality evidence	Fiocchi et al. [232]

**Table 7:** Therapeutic approaches to treatment of allergic disease with microbes and microbial products: studies investigating the utility of helminth therapy on allergic disease outcomes

***Allergic condition***	***Microbial treatment***	***Study outline***	***Follow up period***	***Study outcomes***	***Reference***
Asthma	*Necator americanus*	Thirty-two individuals with asthma and measurable airway responsiveness were randomized and double blinded to cutaneous administration of ten *N. americanus* larvae, or histamine solution (placebo), and followed for 16 weeks	16 weeks	Mean airway hyperresponsiveness improved in both groups, hookworm [1.49 doubling doses (DD)], placebo group (0.98 DD), difference between groups was not significant (0.51 DD; 95% confidence interval: -1.79 to 2.80; P = 0.65)	Feary et al. [237]
Allergic rhinoconjunctivitis	*Necator americanus*	Thirty individuals with allergic rhinoconjunctivitis and measurable airway responsiveness were randomized, double-blind to cutaneous administration of either 10 hookworm larvae or histamine placebo, and followed for 12 weeks	12 weeks	There were no significant differences in peak-flow variability, rhinoconjunctivitis symptoms or skin test responses between groups	Feary et al. [236]
Allergic rhinitis	*Trichuris suis*	100 subjects grass pollen-induced allergic rhinitis randomly administered 8 doses of 2500 live *T. suis* ova or placebo with an interval of 21 days over 24 wks	6 months	No significant change in symptom scores, well days, total histamine, grass-specific IgE, or diameter of wheal reaction on skin prick testing with grass or other allergens tested	Bager et al. [238]
Peanut or tree nut allergy	*Trichuris suis*	18 patients with *T. suis *100—2500 ova orally (dependent on subject age), six doses total with an interval of 14 days over 12 wks		No significant results were reported. There was no change in skin prick test reactivity, except in 1 subject who had a general decrease in reactivity and lost reactivity to peanut	Jouvin and Kinet [239]

Exposure to infections with helminthic nematodes is also largely absent in industrialized societies, raising the prospect for an additional therapeutic avenue to be explored. Parasitic worms or compounds from their excretory/secretory milieu could potentially play a role in mediating tolerance induction either by affecting the composition of organ specific microbiomes or through direct action on the immune system [233]. Infection during early life (0–5 years) with the human parasite *T. trichiuria* has been shown to significantly reduce the incidence of allergy later in life [234], potentially through an early life imprinting of the immune system towards a tolerogenic phenotype, much in the same way that early atopic symptoms can be indicative of initiation of the atopic march. Anecdotal accounts in the news media of individuals self-curing themselves through helminth infections have created much interest in helminthic therapy [233, 235]. However, randomized controlled trials into the efficacy of helminthic treatment for asthma [236], hay fever [237, 238], and nut allergies [239] with either the hook worm *N.americanus* or the whipworm *T.suis* have failed to demonstrate any definitive results. Although, it should be noted that several studies have found success in the use of helminthic therapy for the ongoing treatment of patients suffering from ulcerative colitis [240], indicating that there may indeed be a niche for helminthic therapy in the future of the treatment for allergic disease.

## Summary

With the increased incidence of allergic diseases, a better understanding of the developmental events that leads to immune sensitization against otherwise innocuous environment antigens is a key to the development of rational intervention strategies. The hygiene hypothesis, first put forward over 30 years ago, has now been expanded to include the effects of microbial dysbiosis with the aid of next generation sequencing techniques [205], leading to a much more robust view of how early life exposure to a diverse array of microbes is important for the development of the immune system and the establishment of a homeostatic relationship with our environment [204, 241], especially at the key barrier organs associated with atopy, namely the skin, airways, and gut. The initial premise of the hygiene hypothesis, that a reduction in exposure to viral and bacterial infections during childhood was responsible for the induction of default atopic Th2 responses, has now been largely disproven [199, 201, 202]. Instead, studies demonstrating the importance of the microbiome in modulating the cohort of regulatory cells induced during development which establish a tolerogenic environment and mediate suppression of T cells that arise from inflammatory lineages [148] allows for a better explanation of the observations, that is in parallel to the emergence of hay fever as a “post-industrial revolution epidemic” [179] and the rise of the atopic march [6], that we have also seen a significant increase in the incidence of autoimmunity over the same period.

Multiple studies have described that distinct microbial species found to be associated with either the establishment of a tolerogenic or inflammatory atopic state at the barrier organs. However, the use of probiotics either in a prophylactic measure or to treat established atopic disease has proved to be largely ineffective, so far (Table 1). This may be due to a number of factors including the limited number of probiotics used, the lack of specific targeting of probiotics to individual patients, or the inability of the administered probiotic to reach the specific intestinal niche that would be aided by more precise targeting. Fecal transfer therapies especially those administered by colonoscopy have had great success [215], potentially due to the diverse range of bacteria being transferred from healthy individuals, in addition to avoidance of the stomach environment and enabling access to a broader region of the intestinal system. However, it should be noted that bacteriotherapy systems for oral administration are also being developed and are proving to be effective [242]. Studies are currently underway to assess whether these means of treatment will be effective for atopic disease and the results are eagerly anticipated [216]. An additional avenue which may prove highly useful for future therapy is through the engineering of specific functions into agents for bacteriotherapy. An example of this approach was recently demonstrated in an animal model, where a strain of *Bacteroides* engineered to produce the secondary bile acid isoDCA was introduced and shown to be a potent inducer of gut-associated pTreg cells [167], which can dampen the immune response and support the metabolic function of the gut microbiota [243]. Together, the combined study of the microbiome and the reciprocal relationship of microbes with the development of the immune system has the broad potential for a better understanding of human health and to increase treatment options for atopic diseases, with the hygiene hypothesis proving integral to this path, by shining an initial light on how the microbial dysbiosis prevalent in industrialized societies has affected the regulation of the tolerance inducing mechanisms required for the maintenance of a homeostatic equilibrium with our external environments.
